# Polyester biodegradability: importance and potential for optimisation†

**DOI:** 10.1039/d3gc04489k

**Published:** 2024-03-05

**Authors:** Yue Wang, Robert-Jan van Putten, Albert Tietema, John R. Parsons, Gert-Jan M. Gruter

**Affiliations:** a van ‘t Hoff Institute for Molecular Sciences (HIMS), University of Amsterdam Science Park 904 1098 XH Amsterdam The Netherlands g.j.m.gruter@uva.nl; b Institute for Biodiversity and Ecosystem Dynamics (IBED), University of Amsterdam Science Park 904 1098 XH Amsterdam The Netherlands; c Avantium Support BV Zekeringstraat 29 1014 BV Amsterdam The Netherlands robert-jan.vanputten@avantium.com

## Abstract

To reduce global CO_2_ emissions in line with EU targets, it is essential that we replace fossil-derived plastics with renewable alternatives. This provides an opportunity to develop novel plastics with improved design features, such as better reusability, recyclability, and environmental biodegradability. Although recycling and reuse of plastics is favoured, this relies heavily on the infrastructure of waste management, which is not consistently advanced on a worldwide scale. Furthermore, today's bulk polyolefin plastics are inherently unsuitable for closed-loop recycling, but the introduction of plastics with enhanced biodegradability could help to combat issues with plastic accumulation, especially for packaging applications. It is also important to recognise that plastics enter the environment through littering, even where the best waste-collection infrastructure is in place. This causes endless environmental accumulation when the plastics are non-(bio)degradable. Biodegradability depends heavily on circumstances; some biodegradable polymers degrade rapidly under tropical conditions in soil, but they may not also degrade at the bottom of the sea. Biodegradable polyesters are theoretically recyclable, and even if mechanical recycling is difficult, they can be broken down to their monomers by hydrolysis for subsequent purification and re-polymerisation. Additionally, both the physical properties and the biodegradability of polyesters are tuneable by varying their building blocks. The relationship between the (chemical) structures/compositions (aromatic, branched, linear, polar/apolar monomers; monomer chain length) and biodegradation/hydrolysis of polyesters is discussed here in the context of the design of biodegradable polyesters.

## Abstract 2

To meet EU CO_2_ emission targets, we need to replace most fossil-derived plastics with renewable alternatives. To stop endless pollution by accumulating non degradable plastics we need future plastics to be closed-loop recyclable and/or biodegradable.

## Graphical abstract

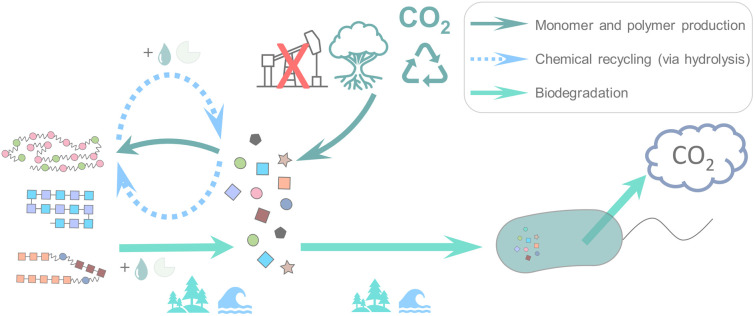

## 1. The problems caused by plastics

Plastics are used for many applications and play a vital role in industry, (food) packaging, textiles, transportation and other uses in daily life. It was reported that the annual production of plastics worldwide was 390 million tons in 2021.^1^ At approximately 40% of the end-use market, packaging represents the largest volume demand for virgin plastics in Europe.^2^ The annual global plastic production is expected to reach 1 billion tons by the end of 2050, with an associated annual CO_2_ footprint predicted at 2.8 billion tons (2.8 Gt).^3^ The increasing production of plastics from fossil-based resources does not conform with targets to reduce global CO_2_ emission. Thus, more sustainable feedstocks, such as biomass, CO_2_ (using carbon capture and utilisation (CCU)) and recycled waste materials, will be required for the next generations of plastics. By changing the feedstock, a substantial portion of the 1 Gt CO_2_ emissions (2023) related to plastics can be avoided and when using renewable energy for the plastics production and a significant amount of CO_2_ for feedstock, it is possible to achieve net-zero emissions.^3–6^

**Fig. 1:**
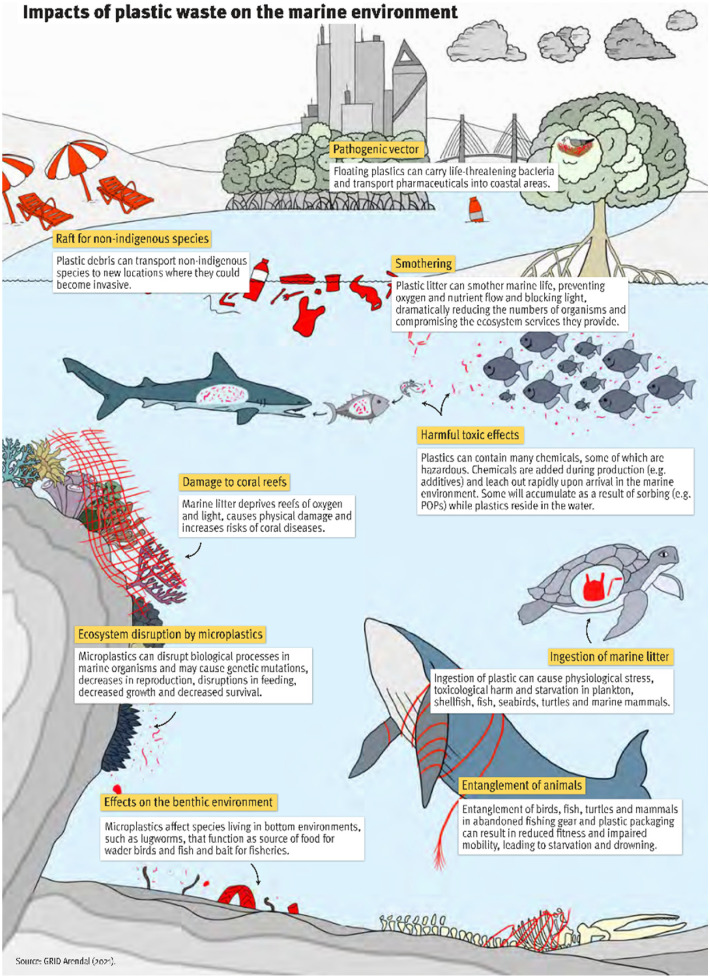
Impacts of plastic waste on the marine environment. Reprinted from GRID-Arendal.^20^

In addition to the impact of plastic manufacturing on climate change, plastic waste is also a severe problem. It is estimated that 6–17 million tons of plastic waste accumulates in the environment annually.^7^ Incorrectly disposed plastic waste leads to environmental pollution and threatens ecosystems (Fig. 1).^8,9^ An illustrative example was presented in the 2019 United Nations Environment Programme report that a pregnant sperm whale washed up dead on a Mediterranean beach with close to 25 kg of plastic waste in her stomach.^10^ Unrecyclable single-use plastic packaging has no post-use value, which is the main cause for plastic pollution, because there is a high risk that these materials will end up in the environment following improper disposal. As conventional plastics are resistant to (bio)degradation, they remain in the environment, and eventually disintegrate into micro- and nanoplastics (MNPs). Prarat and Hongsawat found that microplastics in samples collected from the shore areas in Thailand are mostly polyethylene (PE), polypropylene (PP) and polystyrene (PS).^11^ Additionally, MNPs interact with (hydrophobic) contaminants (as carrier). This raises additional concerns about MNPs as they could contribute to the migration and accumulation of pollutants in the food chain worldwide.^12–17^ For example, in a simple artificial food chain experiment, zebrafish were fed with *Artemia nauplii* (crustaceans) in which very small (1–20 μm) nanoplastics were accumulated with and without benzo[*a*]pyrene as a persistent organic model pollutant (POP).^18^ Microplastics passed the intestinal tracts of zebrafish without significant accumulation and without signs of severe disease even after chronic dietary exposure. The potential of microplastics to act as a vector for POPs was tracked and uptake and distribution within intestinal tracts of zebrafish in histological cryosections was shown. Although problems caused by MNPs in the human body have not been reported yet, the observation of microplastics in the human body raises serious public concern. Recently, plastic particles, including PS, PE and poly(ethylene terephthalate) (PET), were detected and quantified in human blood by Leslie *et al.*^19^ To avoid risks to human health and ecosystems, it is necessary to change our attitude towards plastics and their use and waste-management, and to improve relevant infrastructure.

## 2. Biodegradable plastic: a pollution mitigation supplement to reduce, reuse and recycle

Due to the convenience, low cost and light weight of plastics, it is neither feasible nor sustainable to replace them with other materials for certain applications.^20,21^ For instance, in developing countries, the use of plastic bottled water remains essential for public health with regard to sanitation.^22^ Particularly, the outbreak of COVID-19 raised our reliance on plastic products with the increased production of personal protective equipment, disposable tableware, and packaging waste.^20,23^ Furthermore, plastic packaging improves the shelf-life for food, as well as the efficiency of transportation and distribution, which subsequently decreases food waste, provides consumers with a wider variety of food, and contributes to worldwide food security.^20^ Consequently, plastics are widely considered to be essential for sanitation and food safety.

Recycling of plastic waste is a popular method for addressing the carbon footprint and pollution contributions from plastics.^24^ However, this relies heavily on the infrastructure of solid waste collection systems. In 2020, only 34.6% of Europe's plastic waste was recycled, and this represents the highest level of waste management globally.^2^ Moreover, this value was calculated based on the amount of collection instead of circulation. This demonstrates that even for what is widely considered as high-quality infrastructure for waste management, recycling levels are low, meaning that efficient recycling in the absence of this infrastructure is impossible in less fortunate areas across the globe.

It is not cost-effective to separate complex and contaminated plastic waste with laminated flexible structures (such as food packaging) for conventional mechanical recycling.^25^ Alternatively, chemical recycling can be implemented to convert plastics into smaller molecules, but this is not adopted as commonly as mechanical recycling.^22,25,26^ Currently, only 0.2% of the 34.6% recycled post-consumed plastic in Europe has been chemically recycled.^2^ For chemical recycling, it is important to realise that the circularity potential varies vastly for different plastic materials. While nylons, polycarbonates and polyesters such as PET can be depolymerised to their monomers at very high yields, this is not the case for polyolefins. It is not possible to selectively depolymerise PE and PP respectively back to ethylene and propylene, and a maximum monomer yield of 40–50% is expected from pyrolysis, followed by hydrotreating of polyolefin waste.

Particularly for the packaging segment, which typically holds the highest percentage of recycled plastics, it is estimated that only 14% of plastic packaging is recycled globally, while most packaging plastics are leaked into the environment (32%) or end up in landfills (40%).^27,28^ For applications where single-use plastics are necessary, and collection for recycling is not possible, environmental biodegradability of the plastic should become a required design feature.

The three R's: reduce, reuse and recycle, are a well-known framework to minimise the footprint of plastics. However, as discussed there are limitations, especially in the field of packaging. Therefore, next-generation plastics are expected to be either (1) fully closed-loop recyclable when the relevant infrastructure is available or (2) designed to degrade completely (without the generation of harmful residuals, *e.g.* mineralisation to CO_2_) in the environment over time. Even if a plastic's degradation takes years rather than months, an endless accumulation of plastics into the environment can be prevented, and biodegradable plastics could be used as a supplemental and transitional technology to the three R's framework. (Bio)degradable characteristics may be particularly beneficial for applications with an expected short lifetime and/or when collection would be challenging, for example with (food) packaging, mulch films, fishery materials and disposable medical items.

## 3. Shedding light on biodegradable polyesters

### 3.1 Understanding biodegradation: definitions, mechanisms, and influencing factors

The International Union of Pure and Applied Chemistry (IUPAC) defines a biodegradable polymer as “*a polymer susceptible to degradation by biological activity, with the degradation accompanied by a lowering of its molar mass*”.^29^ This definition emphasises the deterioration of the polymer.

**Fig. 2:**
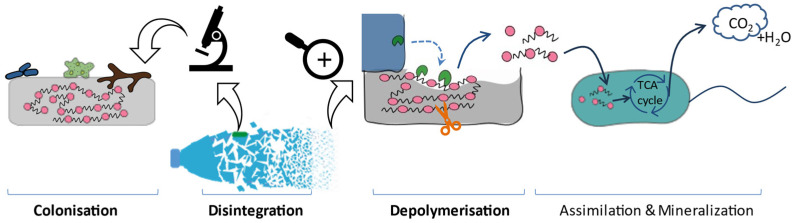
Plastic biodegradation under aerobic conditions. Solid arrows represent carbon flow.^33,38^

Various studies provide slightly different descriptions of biodegradation mechanisms. According to our interpretation, the biodegradation of plastics generally follows these main steps (Fig. 2).^30–37^

• **Colonisation/film formation of microorganisms**: microorganisms can excrete extracellular polymeric substances (EPS, *e.g.* polysaccharides, proteins) to allow them to adhere to the plastic surface.^39^ While some studies consider this to be a crucial initial step in biodegradation, the formation of the film doesn't necessarily indicate that erosion has occurred on the polymeric surface.^40,41^ However, it could have changed the buoyancy of the plastics and enhanced the interactions between hydrophobic and hydrophilic phases, which could affect the following steps indirectly.^33,37,42,43^

• **Disintegration** (macroscopic scale): polymers fragment into smaller size particles due to a combination of independent and interdependent driving forces, including mechanical stress, light, heat, oxygen, water and microorganisms. This leads to alterations in both the physical (*e.g.* morphology, weight loss) and mechanical properties (*e.g.* ductility and tensile strength) of the polymers, and can further-accelerate the formation of MNPs. In addition to these physical processes, such as shearing^44^ and interaction with animals,^36^ disintegration is generally associated with depolymerisation, which can be considered to be the macroscopic result of depolymerisation.

• **Depolymerisation** (molecular scale, *e.g.* hydrolysis for polyesters): the scission of linkages within the polymer chains decreases the molecular weight of the polymer and releases small molecules, including oligomers and monomers, to the environment. Since polymers are too large (high molecular weights) to be taken up by microorganisms, depolymerisation is a prerequisite for the subsequent assimilation stage. The energy required for this cleavage can originate from various sources, such as thermal energy, light energy, mechanical energy, chemical energy, and/or biological energy.^37^ Therefore, certain literature classifies distinct degradation pathways according to the energy source, as extensively discussed in the literature.^36,37,45^ The role of microorganisms is of particular interest, and comprehensive reviews^33,36,46,47^ have covered various types of microorganisms, including bacteria,^48^ fungi,^49^ algae, and their combined interactions,^36^ as well as various enzymes.^50^

• **Assimilation and mineralisation**: microorganisms can take up small molecules released from the previous stage and utilise them as substrate for metabolism and biomass growth.^38^ In this phase, polymer carbon is converted into CO_2_ and biomass under aerobic conditions (or to CO_2_ and CH_4_ under anaerobic conditions), typically *via* the tricarboxylic acid cycle (TCA).^33,49,51^ Generally, these end products resulting from complete biodegradation do not pose an ecological toxicity risk. On the contrary, if depolymerized products cannot be fully assimilated or mineralised, they may present ecotoxic hazard under specific conditions. Microorganisms in this stage may differ from those involved in depolymerisation.^37^

Clearly, disintegration of a polymer is only the beginning of this process and therefore not a good definition for biodegradability. Thus, we recommend the published scientific opinion of the European Union, where biodegradation of plastics is considered as “*the microbial conversion of all its organic constituents to carbon dioxide (CO*_*2*_*) (or carbon dioxide and methane in conditions where oxygen is not present), new microbial biomass and mineral salts, within a timescale short enough not to lead to lasting harm or accumulation in the open environment*”.^52^ This emphasises that the ‘results’ from biodegradation of (biodegradable) plastics should have relatively less environmental impact on nature. The “timescale short enough not to lead to lasting harm or accumulation” is powerful, as this will be very different for each material and for all local circumstances. “Lasting harm and accumulation” are criteria that can be objectively assessed.

Consumers expect that the so-called biodegradable plastics can biodegrade fast in nature without resulting in any negative impact on the environment.^53^ However, some of those commercial plastics claimed to be biodegradable were tested under industrial composting conditions, as described in standard methods ISO 14855,^54^ ASTM D6400,^55^ EN 13432.^56^ The fact that industrial composting provides more favourable conditions (*i.e.* higher temperature, higher total concentration of microorganisms, a higher moisture content for soil) can easily explain that polylactic acid (PLA), a well-known bio-based biodegradable polyester, completely degrades within several months under these conditions, yet only very slow degradation has been observed at ambient temperature.^57–60^ Particularly, the temperature of industrial composting (55–60 °C) could be equal to or above the *T*_g_ of poly(lactic acid) (PLA) (55–62 °C), which leads to the glassy and crystalline polymer becoming softer. This change of state is believed to accelerate the biodegradation rate.^57^ Overall, testing biodegradation at ambient temperature instead of under industrial composting conditions is more representative to evaluate fate-in-nature for biodegradable plastics.

In 2019, Napper and Thompson studied the biodegradation of “compostable” carrier bags. One type completely disappeared within 3 months in a marine environment (note: breakdown into MNPs is not considered to be biodegradation), but minimal loss was observed after 27 months in soil.^61^ Therefore, it is necessary to define the conditions when describing or claiming the biodegradability of plastics. Both internal and external factors, specifically polymer properties and environmental conditions, could significantly influence the primary degradation mechanisms and (bio)degradation rates.

The composition of polymers, *i.e.* structural units and their (sequence) distribution,^62^ plays a decisive role in the properties of the polymer, including the biodegradability (this will be discussed in next section). Separately, other polymer characteristics,^33^ such as hydrophilicity and hydrophobicity,^63^ crystallinity^64^ (*versus* amorphousness), chain flexibility^65^ and orientation, molecular weight and distribution, stereochemical structure, *T*_g_, *T*_m_,^66^ end groups,^67^ free volume,^68^ density, and the presence of residuals, and material properties including morphology, shape, specific surface area, colour^69^ and additives (*e.g.* antioxidants or photosensitizers), could also directly or indirectly affect the (bio)degradation of plastics in the environment. For instance, increased chain flexibility can enhance enzyme accessibility, therefore facilitating hydrolysis.^62,65^

External environmental factors include oxygen availability, UV exposure^44^/weathering, water/moisture/humidity, pH, temperature, salinity, the total amount of microorganisms, and microbial community composition. These factors could directly influence the biodegradation mechanism and rate. They also have indirect effects by shaping microbial communities. For example, salinity has a significant impact on microbial species selection and their metabolic activities.^70^ Other environmental factors like nutrients (C/N ratio) and additional carbon source^71^ could primarily influence this process through their effects on microorganisms.

These factors could interact and combine to influence plastic biodegradation. For instance,^44^ photooxidation can promote the formation of biofilms on the surface of plastics, which, in turn, acts as a shield against UV exposure for the plastic. Furthermore, the biofilm may reduce the buoyancy of the debris in seawater. Subsequently, this accelerates the sinking of debris into unfavourable degradation environments, such as areas with a lower temperature and an absence of light, which may lead to a reduced bioactivity.

### 3.2 Polyesters hydrolysis: mechanisms and advantages

Polyesters, including polybutylene adipate terephthalate (PBAT), PLA, polybutylene succinate (PBS) and polyhydroxyalkanoates (PHA), are the largest group (>67%) of biodegradable thermoplastics produced on industrial scale, followed by starch blends (26%).^4^ PHA is produced by bacteria (natural polyesters) and other synthetic polyesters are typically manufactured *via* esterification polymerisation.

The search for more sustainable feedstocks for next generation plastics can generate a very wide range of building blocks (monomers) for polymer synthesis that are not necessarily available from fossil resources. In particular, biomass and CO_2_ (*via* CCU) can provide a range of non-fossil building blocks (monomers) for polyesters with unique properties; the monomer's chemical structure (composition) largely determines the thermal and physio-mechanical properties of the resulting polymer. It is possible to tune the properties of the resulting polyesters by selecting appropriate building blocks for the required application.^72^ For example, Wang and Gruter reported that they were able to tune the *T*_g_ of a series of fully renewable (co)polyesters poly(isosorbide oxalate-diols) from 60 to 167 °C by varying the isosorbide/diol ratio.^73,74^ Kasmi *et al.* recently reported a potential to tune the *T*_g_ of a series of fully biobased (co)polyesters poly(isosorbide furanoate-*co*-azelate) from −3 to 91 °C by varying the content of 2,5-furandicarboxylic acid (FDCA).^75^

Polyesters can potentially be recycled by both mechanical and chemical recycling processes.^76–79^ Compared to polyolefins, the esterification is a chemically reversible process, and can be reversed *via* hydrolysis or alcoholysis. This means polyesters can be broken down to monomers and these monomers (optionally after purification) can be repolymerised. In this way, the recycling of polyesters is a closed loop process, and downcycling (*e.g.* by mechanical recycling) can be avoided.

**Fig. 3:**
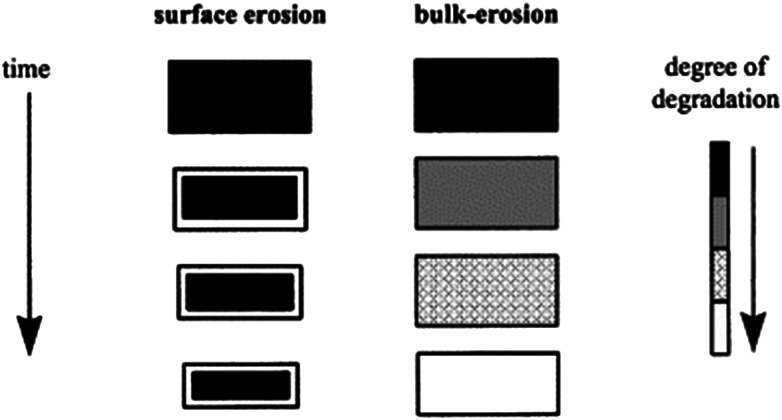
Schematic illustration of the changes a polymer matrix undergoes during surface erosion and bulk erosion. Reproduced from ref. 80 with permission from Elsevier, copyright 2002.

The mechanism of hydrolytic biodegradation for polyesters is described in some studies as a competition between bulk *versus* surface erosion (Fig. 3).^32,80^ The predominant mechanism ultimately depends on the diffusivity of the key reactant (*e.g.*, water or catalyst), the rate of polymer bond cleavage, and the matrix dimensions.^80^ If the diffusivity of the chemical substance throughout the material is faster than the rate of polymer bond cleavage, bulk erosion occurs. This can be observed in the case of abiotic (non-enzymatic) hydrolysis of polyesters. However, when extracellular enzymes are involved in depolymerisation, surface erosion becomes predominant, because the enzymes cannot penetrate the polymer matrix due to their large size. As a result, surface biodeterioration is often mentioned and considered to be an important step in polymer biodegradation. It is important to note that in cases of surface erosion, the material undergoes weight loss, but there is no significant change in the molar mass of the main part of the material. These two mechanisms can also occur simultaneously.

It is also interesting to mention that faster internal degradation (heterogeneous degradation) in PLA and poly(lactic-*co*-glycolic acid) (PLGA). Li provided a detailed explanation of this phenomenon. In brief, it can be explained by the concept of autocatalysis in polyesters.^63^ Autocatalysis refers to the products of a reaction (*i.e.*, hydrolysed carboxylic acids/end-groups) serving as catalysts for the same reaction, thus accelerating the hydrolysis rate of ester bonds.^63^ The hydrolysis products, *i.e.* acids, are trapped inside the polymer matrix rather than being released into solution, subsequently accelerating the hydrolysis rate internally compared to the surface.^63^ The detailed mechanism for acid-catalysed hydrolysis of polyesters is illustrated in Fig. S1. However, Tsuji and Nakahara also reported that adding additional lactic acid (LA) to the solution did not accelerate the hydrolysis of PLA polymer.^81^ Authors attribute this to differences in the availability of free LA in aquatic solutions compared to the polymer matrix.^81^

**Fig. 4:**
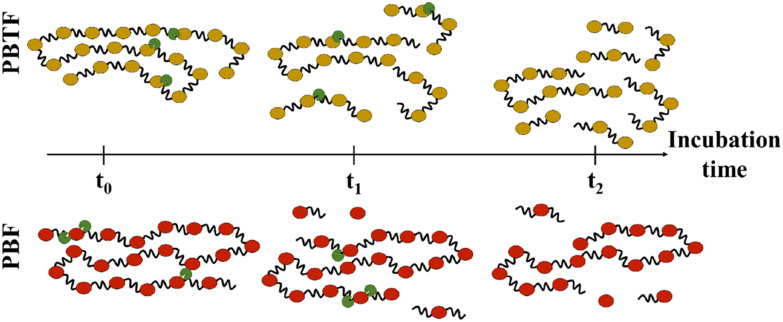
Enzymatic hydrolysis mechanisms. The *endo*-wise hydrolysis mechanism for PBTF (top) leads to the release of oligomers, which are subsequently hydrolysed to monomers. In contrast, the *exo*-wise hydrolysis mechanism for PBF (bottom) leads to the immediate release of monomers. Reproduced from ref. 82 with permission from Elsevier, copyright 2019.

At the molecular scale, the hydrolysis of polyesters can be categorized into two types of scissions: *endo*- and *exo*-scission (Fig. 4). *endo*-Scission refers to the random cleavage of ester bonds along the polyester backbone, resulting in a significant decrease in molecular weight. *exo*-Scission, on the other hand, involves the cleavage of ester bonds at the chain ends, leading to a rather smaller decrease in molecular weight. Different structures and enzymes can lead to different hydrolysis mechanisms. For example, both poly(1,4-butylene 2,5-furandicarboxylate) (PBF, *T*_g_ = 39 °C) and poly(1,4-butylene 2,5-thiophenedicarboxylate) (PBTF, *T*_g_ = 25 °C) can be hydrolysed by cutinase enzymes, but they exhibit *endo*- and *exo*-wise cleavage, respectively. The authors attributed the difference to the lower flexibility/mobility (*i.e.*, higher *T*_g_) of PBF, which limits the access of cutinases and hinders the random scission of ester bonds along the polymer chains.^82^

For the complete biodegradation of a polyester to take place, it first needs to be hydrolysed to small molecules that can be taken up into the bacterial or fungal cell, and in theory, this hydrolysis can occur enzymatically or non-enzymatically. In principle, specific hydrolases (secreted by fungi and bacteria) are required because the rate of non-enzymatic hydrolysis of polyesters is expected to be much slower under mild conditions, such as ambient temperature and/or neutral pH in nature. The occurrence of non-enzymatic hydrolysis is also significantly dependent on the type of ester bonds present in the polyester. Esters from strong carboxylic acids are more easily formed and also are more easily hydrolysed, especially in aquatic environments. When environmental degradability is important, we should opt for polyesters that undergo non-enzymatic hydrolysis as a mechanism for complete (bio)degradation within a reasonable time frame, and especially in aquatic environments. This means that under conditions where biodegradation is disfavoured but where water is present, for example in the deep sea (darkness, cold and low biological activity), non-enzymatic hydrolysable polyesters are suitable to minimise the accumulation of plastics in the environment.

For hydrolysable polyesters, the MNPs could theoretically be hydrolysed more easily than macroplastics due to relatively larger surface areas. The breaking down of oligomers and monomers will continue in the presence of moisture. If the hydrolysis products are known to be biodegradable and not harmful, then full environmental biodegradation is assured. It is also important to realise that breakdown to monomers and small oligomers is slow and will be diluted in nature, which will mitigate risks of monomers that have some toxicity (at elevated concentration) for bacteria and fungi.

Compared to current packaging material (mainly PP, PE, PET),^1^ hydrolysable plastics can reduce the lifetime of microplastics, which in-turn reduces the likelihood that they absorb and spread contaminants into the food chain. Therefore, hydrolysable polyesters may also contribute to minimising the negative effects of MNPs.

## 4. Structural effects on the hydrolysis and biodegradation rate of polyesters

Both chemical structures and physical properties, including molecular weight, glass transition and melting temperature (*T*_g_ and *T*_m_), crystallinity, rigidity and hydrophilicity, impact the potential for polyesters to biodegrade within a reasonable time frame in various environments. The chemical structure is a major factor in determining thermomechanical and most physical properties of polyesters. As a consequence, (co)polymerisation of different monomers leading to varying compositions is a commonly employed strategy to tune the final properties of polyesters and make them suitable for certain applications. Biodegradability of polyesters is also expected to be tuneable *via* the same strategy. Therefore, by making copolyesters, it is possible to design biodegradable/hydrolysable polyesters with favourable high thermal- and good mechanical properties.

Understanding how the structure of polyesters affects their biodegradability is important for the development of novel plastics. Therefore, in this section, the impacts of the chemical structure on biodegradation or hydrolysis of polyesters are discussed by presenting specific selected examples (a summary table is provided in the ESI, Table S1†). For a detailed discussion on how thermal and mechanical properties are effected by the chemical structure of polyesters, see Larrañaga and Lizundia^72^ and references mentioned below.

The conventional polyesters, such as PET, exhibit decent thermal and mechanical properties with their high content of the terephthalic acid (TPA) aromatic building block. However, the TPA polyesters are resistant to hydrolysis and biodegradation, as discussed in detail in the TPA section below. Consequently, strategies aimed at enhancing the biodegradability of conventional polyesters involve incorporating monomers that form ester bonds that more easily hydrolyse, such as aliphatic esters, monomers with more acidic carboxylic acids such as oxalic acid or blending them with biodegradable polyesters. It's important to note that the latter approach is not recommended due to the risk of non-biodegradable polyesters persisting and forming MNPs.

Most commercially available biodegradable polyesters, including PLA, PBS and polyhydroxybutyrate (PHB), are aliphatic polyesters with linear structures. However, they generally possess unfavourable thermal- and/or mechanical properties for many applications. The incorporation of rigid aromatic and (bi)cyclic monomers, like terephthalate, furan dicarboxylate, isosorbide, cyclobutene diol or cyclohexane dimethanol into polyesters typically improves their thermal and mechanical properties. Therefore, copolyesters with rigid (bi)cyclic co-monomers attract a lot of attention, but their synthesis is often challenging.

### 4.1 Aromatic diacid monomers

#### Terephthalic acid (TPA)

Among various aliphatic–aromatic copolyesters, the most popular is poly(butylene adipate-*co*-terephthalate) (PBAT, structure in Fig. 7), which is commercially available and accounts for 19.2% of the biodegradable plastic market.^4^ Meanwhile, extensive research is being done on improving its properties and understanding its mechanism of (bio)degradation.

**Fig. 5:**
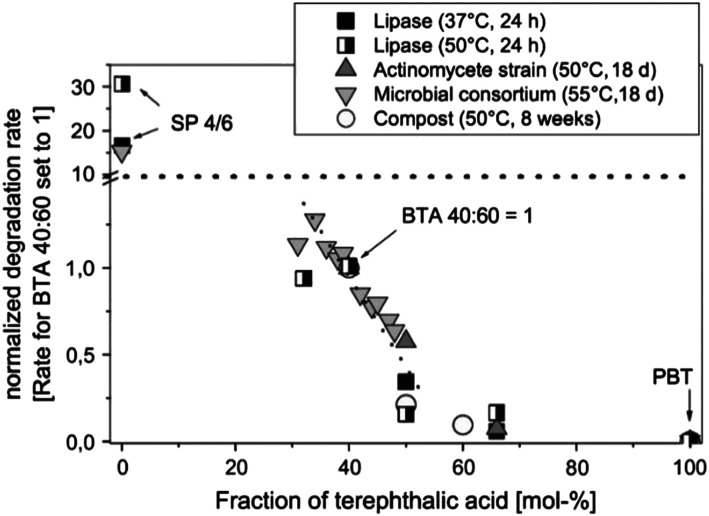
Dependence of the degradation rate of aliphatic–aromatic copolyesters (BTA-copolyesters, PBAT) on the content of terephthalic acid in the polymer in different degradation environments. The absolute degradation rates are normalised to the rate for the random copolyesters BTA_random_ 40 : 60. B: 1,4-Butanediol unit; T: terephthalic acid unit; A: adipic acid unit; SP 4/6: poly(butylene adipate) (SP 4/6). Reproduced from ref. 62 with permission from Elsevier, copyright 2005.

It as has been reported widely that higher terephthalate content may reduce the enzymatic degradation rate of copolyesters. When the terephthalate content is above 60 mol% (relative to the total diacid), copolyesters are considered non-biodegradable, similar to purely aromatic polyesters (Fig. 5).^62,83^ This reduced biodegradability can be attributed to the limited accessibility of the enzyme to catalyse the cleavage of ester bonds, likely caused by the aromatic groups.

**Fig. 6:**
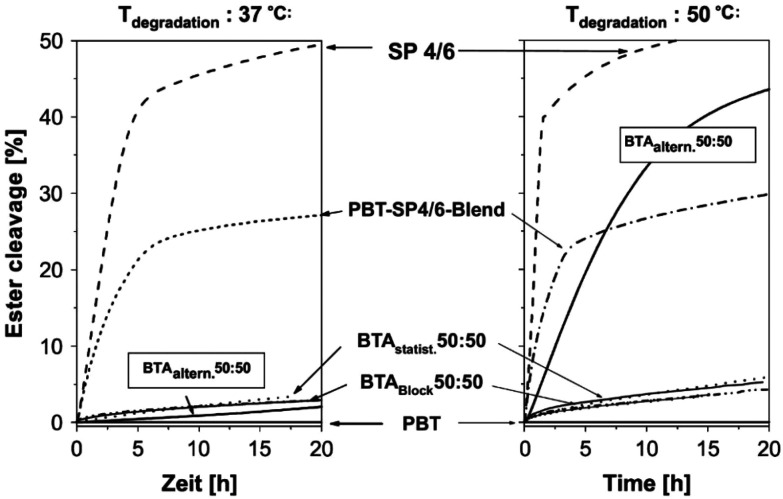
Degradation of polyesters of different micro-structure by a lipase from *Pseudomonas* sp. (PsL) at 37 °C and 50 °C in water. Degradation was monitored by titrating the acid groups formed during ester cleavage. B: 1,4-Butanediol unit; T: terephthalic acid unit; A: adipic acid unit; SP 4/6: poly(butylene adipate) (SP 4/6). Reproduced from ref. 62 with permission from Elsevier, copyright 2005.

Interestingly, Marten *et al.* studied the enzymatic hydrolysis of PBAT50/50 with different micro-structures, including random and strictly alternating, at 37 and 50 °C respectively (Fig. 6).^62^ It is found that neither of them were significantly hydrolysed at 37 °C, while the alternating one hydrolysed at 50 °C. Even though the molecular weight of the alternating PBAT was only about one third of the other (18 and 51 kg mol^−1^), this difference was attributed to their different *T*_m_ (random, 132 °C; alternating, 85 °C), which also gave an indication of the mobility of the chains. The authors suggested that the chain mobility of polymers increased with an increase in the temperature of the environment, which allows the access by lipase (with a deep active site) for the alternating PBAT50/50 (BTA_altern._ 50 : 50) at 50 °C but not for the random copolyesters (BTA_statist._ 50 : 50). Therefore, they concluded that it may not be possible to design polyesters with high *T*_m_ combined with high degradation rate at ambient temperature, unless polyesters could degrade *via* mechanisms other than enzymatic hydrolysis by lipase (deep active site). This means that polyesters with high *T*_m_ could be hydrolysed either non-enzymatically or by other enzymes, for instance enzyme with shallow active sites as shown in the next paragraph.

**Fig. 7:**
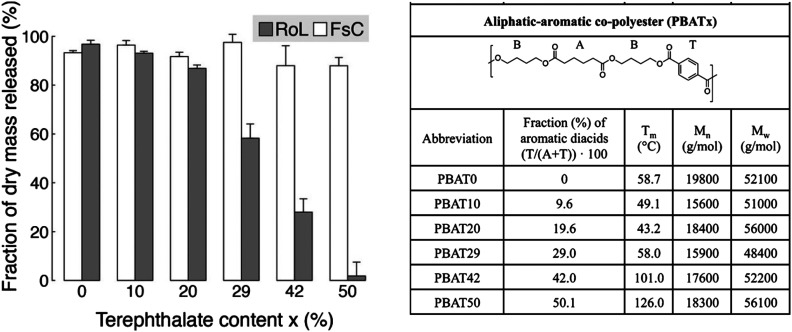
Hydrolysis of poly(butylene adipate-*co*-terephthalate) (PBATx) thin films by *Fusarium solani cutinase* (FsC) and *Rhizopus oryzae lipase* (RoL) at pH 6 and 20 °C, as measured with a quartz-crystal microbalance with dissipation monitoring (QCM-D). The different PBATx polyesters varied in the molar fraction of the aromatic diacid terephthalate (T) to the aliphatic diacid adipate (A) (*i.e.*, *x* = T/(A + T) × 100). Fraction (%) of initially coated dry polyester mass that was released during the hydrolysis experiments. Error bars represent deviations of duplicate measurements from their mean. Reproduced from ref. 65 with permission from American Chemical Society, copyright 2017.

Moreover, Zumstein *et al.* investigated the relationship between chain flexibility of PBAT (dependent on the ratio of aromatic and aliphatic monomers) and the active site of the enzyme.^65^ Enzymes with a deep active site (*e.g. Rhizopus oryzae lipase* (RoL)) require higher chain flexibility to hydrolyse PBAT, while *T*_m_ (43–126 °C) has little effect on enzymes with shallow active sites (*Fusarium solani cutinas*e (FsC)) (Fig. 7).

#### Furan dicarboxylic acid (FDCA)

**Fig. 8:**
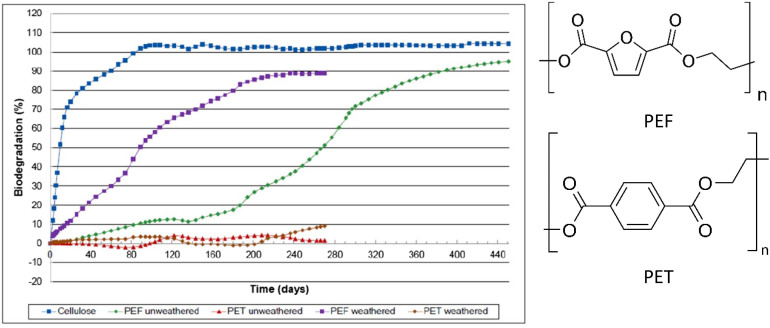
Biodegradation profiles of weathered and un-weathered PEF, as well as weathered and un-weathered PET and cellulose as a reference material. Biodegradation (%) = amount of polymer converted to CO_2_ (up to 450 days) with air/oxygen @ 58 °C in soil. All curves are the averages of three samples.^85^

The only aromatic compound identified by the US Department of Energy in the twelve sugar-based future building blocks was 2,5-furandicarboxylic acid (FDCA).^84^ One of it's derived polymers, polyethylene 2,5-furandicarboxylate (PEF), is considered a promising alternative for PET, because terephthalic acid today is only available from fossil resources. PEF has high-potential applications in packaging as it has better mechanical, thermal (higher *T*_g_) and gas barrier properties than PET. Moreover, PEF was reported to be industrially compostable, while PET is not (Fig. 8).^85^ The biodegradation of PEF under ambient conditions is under investigation.

**Fig. 9:**
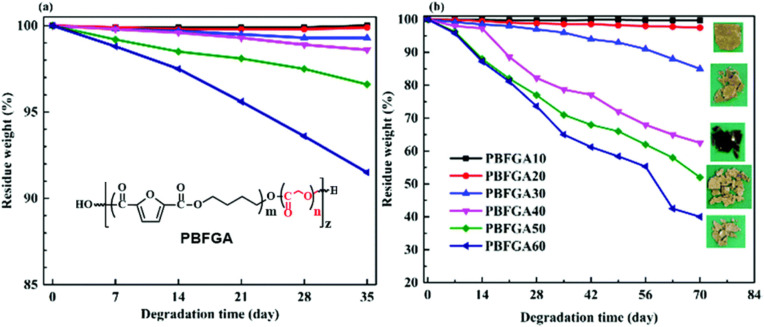
Weight loss curves of PBFGAs during hydrolysis (a), enzymatic degradation (b) and visual observations of degraded films after 70 d at 37 °C. Note: Scales are not equal for (a) and (b). Reproduced from ref. 86 with permission from the Royal Society of Chemistry, copyright 2019.

However, much like TPA, aromatic-aliphatic copolyesters with higher FDCA content were reported to hydrolyse relatively slowly at low temperature (non-industrial composting). For instance, Hu *et al.* synthesised a series of poly(butylene furandicarboxylate-*co*-glycolate, PBFGA) copolyesters with different molar ratios of glycolate (GA) and furanoate to conduct hydrolysis experiments with and without *porcine pancreas lipase* in phosphate-buffered saline at 37 °C.^86^ Weight loss was observed for PBFGAs with more than 30 mol% GA units (with fully random micro-sequential structures) after 35 days, independent of the presence of enzyme. For PBFGA20, slight weight loss, with visible holes in the surface, was observed for lipase only after 70 days. Higher contents of FDCA further decreased the hydrolysis rate of PBFGA copolyesters (Fig. 9). The authors suggested that the steric hindrance of the furan ring restrains hydrolysis. The threshold of FDCA content for biodegradability is likely higher than for TPA.

It is important to note that *T*_g_'s of all PBFGAs are around 37 °C (Table 1), and the experiments were performed at 37 °C. The low *T*_g_'s indicate the polymer chains have a more flexible structure than for instance PLA (*T*_g_ 55–62 °C) or PET (*T*_g_ > 70 °C). The faster degrading compositions (40% GA content and higher) were all amorphous, whereas those with less GA exhibited increasing levels of crystallinity with decreasing amounts of GA, resulting in reduced degradation. This suggests that a low *T*_g_ (*i.e.* close to the temperature of degradation) combined with an amorphous structure likely makes the ester bonds more accessible for both water and enzymes.

**Table 1:** Thermal properties of PBFGAs. Reproduced from ref. 86 with permission from the Royal Society of Chemistry, copyright 2019

	1st heating scan		1st heating scan after annealing		2nd heating scan		
	*T*_m_ (°C)	Δ*H*_m_ (J g^−1^)	*T*_m_ (°C)	Δ*H*_m_ (J g^−1^)	*T*_g_ (°C)	*T*_m_ (°C)	Δ*H*_m_ (J g^−1^)
PBFGA60	nd	nd	nd	nd	37.7	nd	nd
PBFGA50	nd	nd	nd	nd	37.6	nd	nd
PBFGA40	nd	nd	84.5	2.7	37.3	nd	nd
PBFGA30	110	0.6	112.8	17	36.3	nd	nd
PBFGA20	138.7	14.1	141.7	31	38.2	nd	nd
PBFGA10	158.7	24.1	156	33.2	37.2	155.6	26.7
PBF	168.3	34.8	167.8	37.5	36.9	170.3	30.8

### 4.2 Cyclic aliphatic diol monomers

#### Isosorbide

As well as aromatic compounds, cyclic aliphatic diols have also been used for improving thermal properties of polyesters. Isosorbide (IS) is one of these bio-based diols, and is commercially produced at about 20 000 ton per year by Roquette in France.

**Fig. 10:**
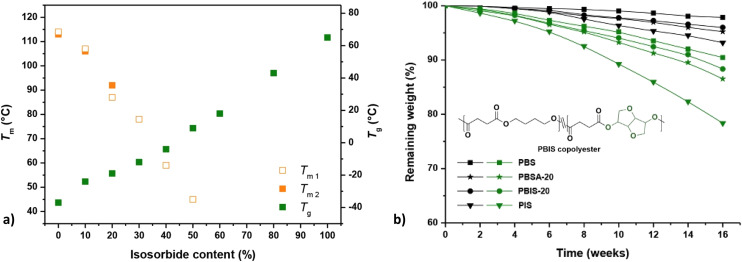
Melting and glass transition temperatures of PBIS copolyesters as a function of isosorbide contents (a). Melting temperatures were obtained during the first and the second heating scans using differential scanning calorimetry (DSC) (*T*_m1_, *T*_m2_). Enzymatic degradation of polyesters (b). Remaining weight of samples tested with (green) or without (black) the presence of *porcine pancreas lipase* at 37 °C (b). Note: PBSA-20 (A = adipate): *T*_m1_ = 98 °C, *T*_m2_ = 97 °C, *T*_g_ = −42 °C. Reproduced from ref. 87 with permission from Elsevier, copyright 2017.

Qi *et al.* incorporated isosorbide into PBS and obtained a series of random copolymers (PBIS) with a wide range (0–100%) of IS content.^87^ It was found that the increase in isosorbide almost linearly improves the *T*_g_ but reduces the crystallinity and *T*_m_ of the resulting copolyesters (Fig. 10a). This was attributed to the molecular structure of isosorbide, which is rigid and asymmetric. Similar effects on *T*_g_ were also observed by van der Maas.^88^ Modification of these properties (*i.e. T*_g_, *T*_m_, crystallinity) are also expected to affect the biodegradation behaviour of PBIS copolyesters.

In the same study, Qi *et al.* compared hydrolysis of homopolymers (PBS and PIS) and copolymers PBIS-20 (20 mol% isosorbide) and PBSA-20 (20 mol% adipate).^87^ The incorporation of isosorbide was found to facilitate the hydrolysis, especially for enzymatic hydrolysis with *porcine pancreas lipase* and for the homopolymer PIS (higher content of isosorbide, Fig. 10b). This was attributed to the increased hydrophilicity/hygroscopicity and reduced crystallinity resulting from isosorbide. However, the *T*_g_'s of PBS and PBIS-20 were lower than the experiment temperature (37 °C), while the *T*_g_ of PIS was rather higher. Together with steric hindrance caused by isosorbide, these factors are actually expected to impede enzymatic hydrolysis of polyesters containing isosorbide.

**Fig. 11:**
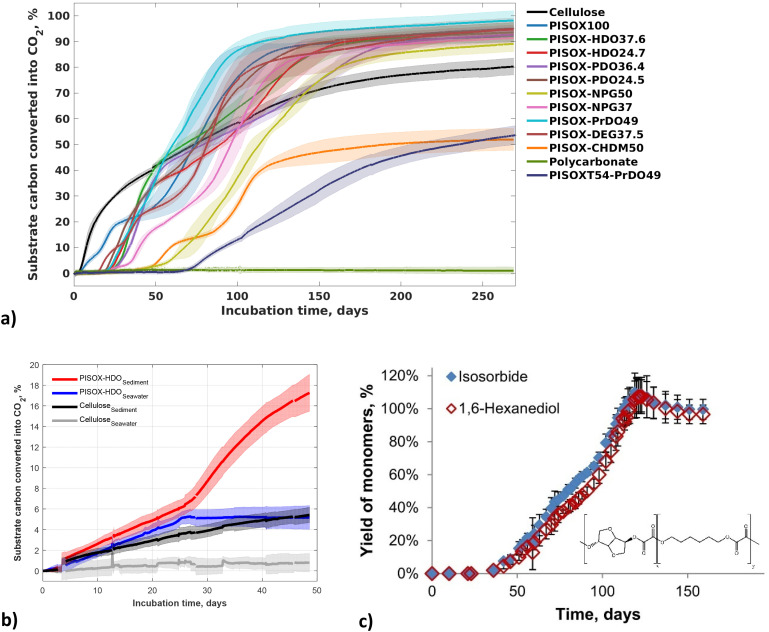
Biodegradation curves of PISOX, PISOX copolyesters, cellulose and polycarbonate (positive and negative reference) with approximately 5 mg (substrate) carbon per gram of dry soil at 25 °C (a). Biodegradation curves of PISOX-HDO25 and cellulose in seawater and on the interface of seawater and sediment (b). Mean biodegradation percentages (lines) were plotted. The shaded area represents the standard deviation of at least three replicates. Hydrolysis of PSIXO-HDO25 (individual yield monomers: isosorbide and 1,6-hexanediol) during 6-month hydrolysis at 25 °C in D_2_O as percentage of their theoretical maximum release (c). Error bars represent standard deviation of triplicate hydrolysis experiments.^60,89^

In addition, we reported the (bio)degradation of poly(isosorbide-*co*-diol oxalate) (PISOX), a new class of high *T*_g_ (tuneable *T*_g_'s of sub-zero to 167 °C) renewable (co)polyesters at ambient temperature (25 °C) in soil as well as their non-enzymatic hydrolysis (Table 2 and Fig. 11a).^89^ This novel material was designed to combine high *T*_g_ (>100 °C) with good biodegradation. A representative of copolyesters poly(isosorbide-*co*-1,6-hexanediol) oxalate with 75/25 mole ratio isosorbide/1,6-hexanediol demonstrated relatively fast biodegradation in soil as well as in the marine environment and the relationship between biodegradation and the non-enzymatic hydrolysis was discussed in our earlier study (Fig. 11b and c).^90^

**Table 2:** Overview of the composition and thermal properties of previously evaluated PISOX copolymers^89^

Polymer	% IS relative to total diol	Co-monomer	Co-monomer structure	% co-diol relative to total diol	*T*_g_, °C
PISOX100	100.0%	—	—	—	167
PISOX-DEG37.5	62.5%	Diethylene glycol, DEG	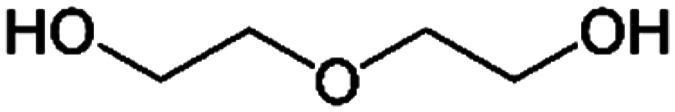	37.5%	88
PISOX-PrDO49	51.0%	1,3-Propanediol, PrDO	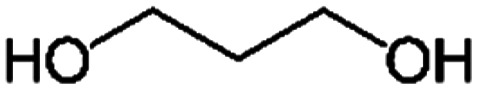	49.0%	85
PISOX-PDO36.4	63.6%	1,5-Pentanediol, PDO	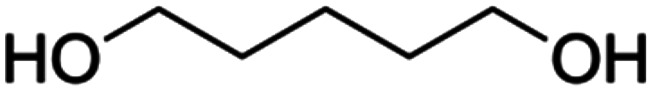	36.4%	85
PISOX-PDO24.5	75.5%	1,5-Pentanediol, PDO		24.5%	110
PISOX-HDO37.6	62.4%	1,6-Hexanediol, HDO	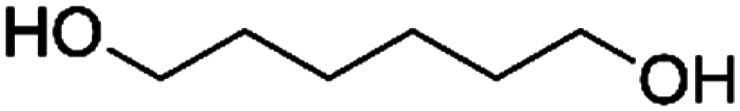	37.6%	76
PISOX-HDO24.7	75.3%	1,6-Hexanediol, HDO		24.7%	107
PISOX-NPG50	50.0%	Neopentyl glycol, NPG	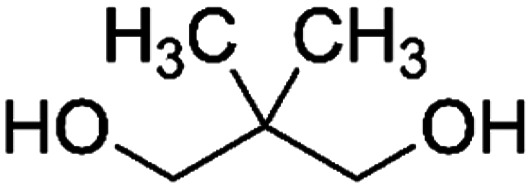	50.0%	83
PISOX-NPG37	63.0%	Neopentyl glycol, NPG		37.0%	102
PISOX-CHDM50	50.0%	1,4-Cyclohexanedimethanol, CHDM	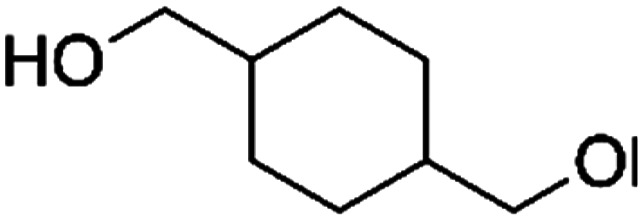	50.0%	101
PISOXT54-PrDO49	51.0%	1,3-Propanediol	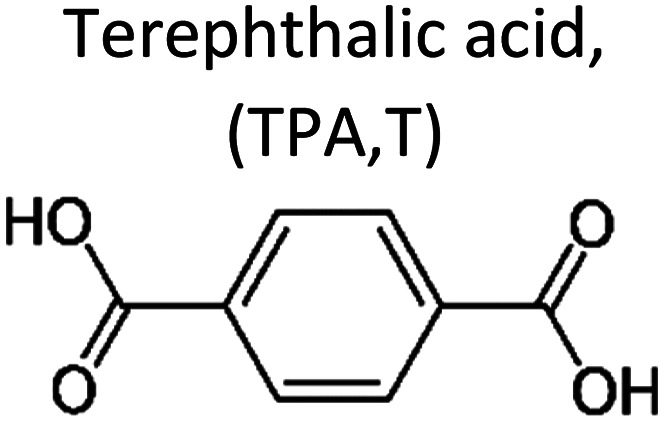	49.0%	102
	46%^a^	Terephthalic acid		54%^b^	

a % oxalic acid relative to total diacid.

b % TPA relative to total diacid.

In this study,^89^ all (co)polyesters with non-cyclic co-monomers biodegraded completely within 6 months in soil (Fig. 11a). They show biodegradability comparable to (or even better than) that of cellulose under the same conditions. The PISOX copolymers with 1,4-cyclohexanedimethanol and terephthalic acid as third and fourth comonomer did not exhibit fast biodegradation. However, the PISOX homopolymer, which has a similar content of cyclic rigid monomers, degraded the fastest (Fig. 11a). This indicates that isosorbide, as a biobased rigid cyclic monomer, can provide good thermal (*i.e. T*_g_) and mechanical properties of polymers without sacrificing biodegradability when combined with oxalic acid.

Resistance to heat (*i.e.* high *T*_g_) for amorphous polymers is generally considered an unfavourable factor for facile environmental biodegradability. An increased difference between *T*_g_ and the temperature of the environment negatively affects the mobility of the polymer (like the *T*_m_), and has been attributed to limiting the access to the enzyme, especially for those enzymes with deep active sites. This appears to contrast the biodegradation of PISOX copolyesters, but we saw that non-enzymatic hydrolysis of the oxalate building blocks allowed environmental biodegradability despite the materials having very high *T*_g_'s.^90^

### 4.3 Linear aliphatic monomers

**Fig. 12:**
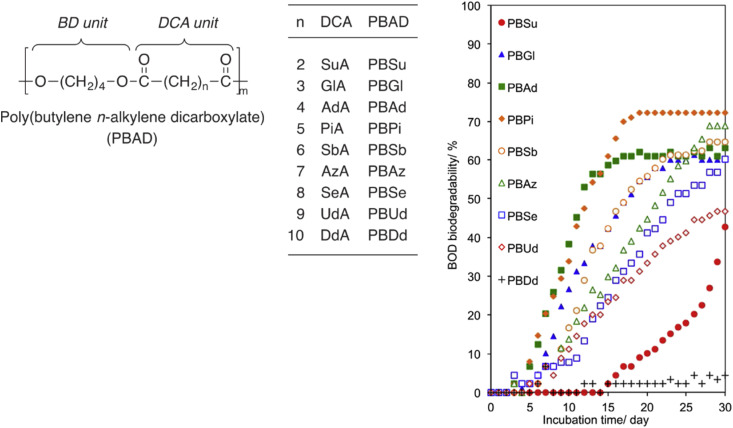
BOD biodegradation curves of PBADs by inoculum mixture of four soils at 25 °C for 30 days. Reproduced from ref. 91 with permission from Elsevier, copyright 2017.

In general, the chain length, or the number of methylene units, of linear aliphatic building blocks is expected to affect thermal–mechanical properties of polyesters as well as biodegradability. To systematically investigate the effect of the number of methylene units on biodegradability of linear polyesters, Baba *et al.* conducted polycondensations of 1,4-butanediol with nine aliphatic α,ω-dicarboxylic acids (C4–C16) to obtain PBADs (Fig. 12).^91^ Their environmental biodegradability was determined by biochemical oxygen demand (BOD, *i.e.* oxygen consumptions) in water (inoculum mixture prepared with 4 soils) at 25 °C. After 30 days, it was found that PBDd (C12) barely biodegraded, and biodegradation rates of PBUd and PBS(u) (C11, C4) were slower than the others (Fig. 12). Despite the long lag phase of PBS(u) and limited incubation time, biodegradability of PBS(u) (during a longer period) could be considered comparable with others, taking into account the steep upward trend at the end of incubation. These results suggested that medium chain length (C4–C8) linear aliphatic building blocks had a similar effect on the biodegradability of polyesters, which is in line with our observations for PISOX copolyesters (Fig. 11a),^89^ while long chain length diols had a negative effect.

**Fig. 13:**
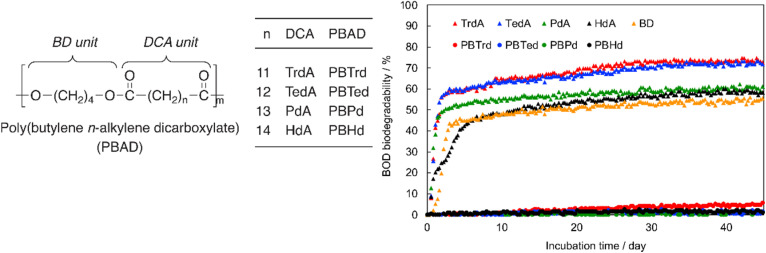
BOD biodegradation curves of 1,4-butanediol (BD), *n*-alkylene dicarboxylic acid (DCAs), including tridecanoic diacid (*n* = 11; TrdA), tetradecanoic diacid (*n* = 12; TedA), pentadecanoic diacid (*n* = 13; PdA), and hexadecanoic diacid (*n* = 14; HdA), and PBADs at 25 °C for 45 days. Reproduced from ref. 92 with permission from Elsevier, copyright 2021.

Tachibana *et al.* also conducted the BOD-test for PBADs with longer chain dicarboxylic acids (C13–C16, Fig. 13).^92^ As expected, quite limited biodegradation (0–5%) was reported after 45 days.

**Fig. 14:**
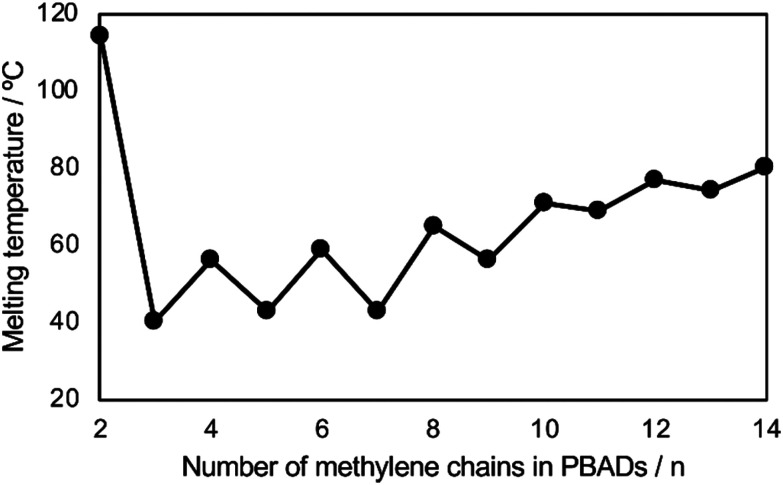
Relationships of *T*_m_ of PBAD and number of methylene (*n*) in the alkylene dicarboxylate of PBADs. Reproduced from ref. 92 with permission from Elsevier, copyright 2021.

The even–odd effect on *T*_m_ was demonstrated clearly with these PBADs (Fig. 14).^92^ However, this effect did not appear to apply to their biodegradability. Additionally, *T*_g_ of PBADs (<−16 °C) are far lower than the incubation temperature.

**Fig. 15:**
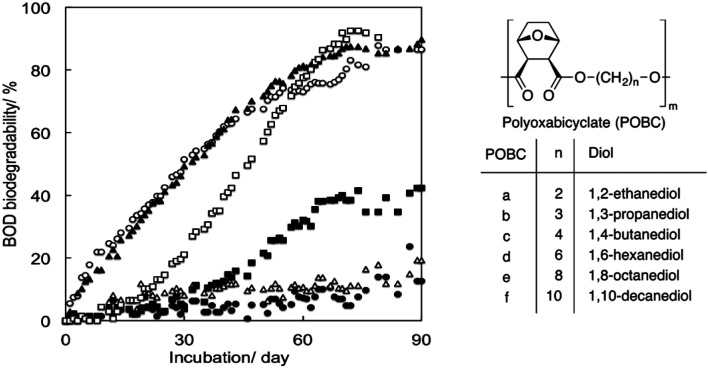
BOD biodegradation curves of POBCs (●: POBC-a, ○: POBC-b, ▲: POBC-c, △: POBC-d, ■: POBC-e, and □: POBC-f) by the mixture inoculum at 25 °C. Reproduced from ref. 93 with permission from Elsevier, copyright 2017.

Tachibana *et al.* polymerised oxabicyclodicarboxylic anhydride (OBCA, derived from furfural) and six linear diols of varying chain length units (Fig. 15).^93^ The same method from the previously-mentioned study was used to assess biodegradability. Generally, no clear effect of the chain length of the diol units on biodegradability could be observed. Three compositions, *i.e.* diols with chain lengths of 3, 4 and 10 carbon atoms in polyoxabicyclates (POBC), showed high biodegradability in the BOD tests, while POBCs with C2 and C6 showed much lower biodegradability after 90 days. There is therefore no direct relationship between the chain length of the diol and the biodegradability of the polyester. Considering the rapid mineralisation (under the same conditions) of all the diol monomers, the rate-limiting step should be the hydrolysis of the polymers by enzymes produced by the microorganisms in the inoculum. Additionally, in comparison with PBADs, this study showed that incorporation of cyclic building blocks delayed biodegradation significantly.

#### Oxalic acid

Oxalic acid is particularly interesting as a rigid diacid that can potentially be obtained from CO_2_.^94^ Polyesters from oxalic acid (polyoxalates), containing two ester functional groups adjoined directly show susceptibility to non-enzymatic hydrolysis.^90^ Currently, attention is mainly focused on medical applications, such as drug carriers. A table summarising the hydrolysis results can be found in the ref. 90.

Therefore, polyoxalates are expected to lead to fast biodegradation in various environments. Combined with isosorbide, poly(isosorbide-*co*-diol oxalate) polyesters (PISOX-diol) were developed, which demonstrate good mechanical properties, good water vapour- and oxygen barriers, and good thermal properties. Their biodegradation is discussed in Section 4.2 (Fig. 11). Additionally, non-enzymatic hydrolysis of these polyesters was performed at 25 °C in the same study, copolyesters with linear co-diol were non-enzymatically hydrolysed completely within 180 days.^89,90^ This indicates that facile hydrolysis of oxalate esters is essential for the fast biodegradability of PISOX (co)polyesters at ambient temperature in soil.

Furthermore, the presence of oxalate in polyesters was shown to favour relatively fast biodegradation, independent of the type of the non-cyclic co-diol, such as 1,3-propanediol, diethylene glycol, 1,5-pentanediol, 1,6-hexanediol and neopentyl glycol. Similar to the study of Baba *et al.*, no obvious trend was observed for the chain length of the diol (C3–C6) on the biodegradability of PISOX copolyesters.^89,91^

### 4.4 Branched monomers

#### Poly(lactic-*co*-glycolic acid) (PLGA)

PLA was the largest bio-based plastic produced at an industrial level in 2020, mainly for packaging and disposable tableware applications.^95^ Lactic acid (LA) is industrially produced by the fermentation of glucose. Glycolic acid (GA) can also be made from biomass, but potentially also from CO_2_.^96^ LA/GA copolymer, poly(lactic-*co*-glycolic acid) (PLGA), is one of the most widely investigated biodegradable and biocompatible copolyester for controlled release devices.^97^ Relatively fast hydrolysis of PLGA copolyesters *versus* very slow hydrolysis of PLA effectively demonstrates the negative effect of the methyl branch or side chain of lactic acid on the hydrolysis rate by increasing the hydrophobicity.

**Fig. 16:**
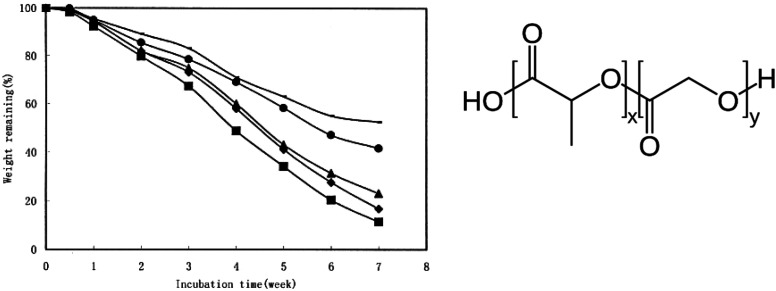
Weight remaining percentage of (**—**) PLA, (●) PLGA (85/15), (▲) PLGA (75/25), (●) PLGA (65/35), and (■) PLGA (50/50) microspheres containing human serum albumin incubated in phosphate-buffer saline (154 mM, pH 7.4) at 37 °C. Each point represents the mean of three individual samples of microspheres. Reproduced from ref. 98 with permission from John Wiley & Sons Inc., copyright 2003.

Specifically, varying the ratio between LA and GA allows for tuning the sensitivity to hydrolysis for PLGA. For example, Zhou *et al.* presented the non-enzymatic hydrolysis profile of a series of PLGAs with 50% to 100% molar content of LA in phosphate-buffered saline at 37 °C (Fig. 16).^98^ The losses of microsphere (containing human serum albumin) weight and polymer intrinsic viscosity for PLGAs in a 7-week hydrolytic incubation were presented. Both rates decreased with the increase in LA molar ratios in PLGA in spite of slight increase in *T*_g_.

The negative impact of LA monomers on hydrolysis for PLGA (LA > 50%) was also reported in other studies.^67,97,99^ This was attributed to the lower hydrophilicity of LA *versus* GA. The methyl branch/side-chain makes the lactate ester groups more sterically hindered than the glycolate ester groups, which could reduce the accessibility to water.

**Fig. 17:**
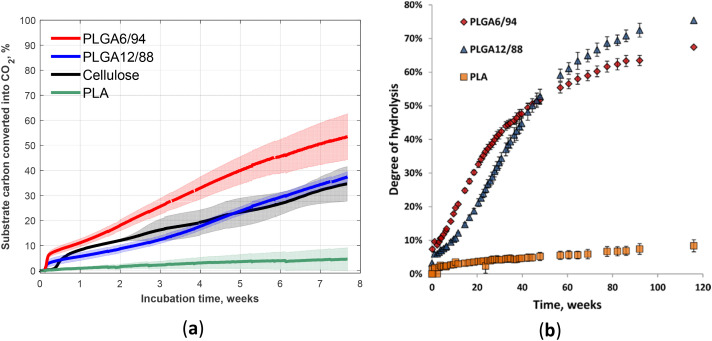
Fifty-three-day biodegradation curves of PLA, PLGA12/88, PLGA6/94 and cellulose (reference) at 25 °C in soil (a). Mean biodegradation (lines) were plotted. The shaded area represents the standard deviation of replicates. Degree of hydrolysis for PLGA6/94, PLGA12/88 and PLA *versus* time over 116 weeks at 25 °C in D_2_O (b). The points represent the averages of triplicate experiments, with the error bars representing the standard deviation.

The biodegradation and hydrolysis of relatively novel PLGA with high GA content (PLGA12/88 and PLGA6/94) was studied (Fig. 17).^60^ These polymers biodegraded at a rate similar to cellulose (the reference), which reached approximately 40% mineralisation within 53-day incubation in soil (Fig. 17a).^60^ However, the non-enzymatic hydrolysis was not that fast (incomplete over 800 days, Fig. 17b).^60^ The comparison of the hydrolysis profile of these two compositions suggested the competition of two factors that determine the relative hydrolysis rate of these PLGA copolymers: on the one hand, higher LA content results in less hydrophilicity, primarily affecting the early stages of hydrolysis; on the other hand, the presence of crystalline areas with higher GA content appears to slow down the hydrolysis in the later stages.^97^

#### Polyhydroxyalkanoates (PHA)

**Fig. 18:**
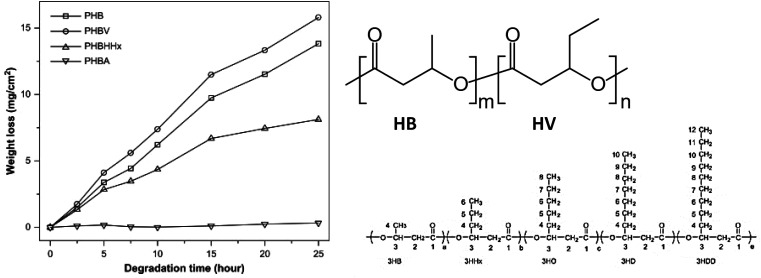
Enzymatic degradation of PHBV (94%), PHB (75%), PHBHHx (39%) and PHBA (0%) by PHA depolymerase produced by *Ralstonia pickettii* T1 at 37 °C. Structures of monomers and PHBV (*m*-HB, *n*-HV, as an example for copolymers). Reproduced from ref. 100 with permission from Elsevier, copyright 2007.

Side-chains with increasing side-chain length can hinder enzymatic hydrolysis of copolyesters more. For example, Li *et al.* compared the enzymatic hydrolysis by PHA depolymerase produced by *Ralstonia pickettii* T1 of several microbial biopolyesters, polyhydroxyalkanoates (PHAs), consisting of 3-hydroxybutyrate (HB) and different chain-length 3-hydroxyalkanoates within 25 hours at 37 °C (Fig. 18).^100^Table 3 provides an overview of the PHAs tested, with a description of their composition.

**Table 3:** Compositions (see also Fig. 18), molecular weights and thermal properties of PHB, PHBV (19.1%), PHBHHx (18.8%) and PHBA (15.2%) used in ref. 100. Structures of monomers, HB, HV, HHx, HO, HD and HDD see in Fig. 18. Reproduced from ref. 100 with permission from Elsevier, copyright 2007

Sample	P(3HA) compositions (mol%)			Molecular weight		Thermal properties		
	HB	Other monomer	HD, HDD	*M*_n_ (×10^5^)	*M*_w_/*M*_n_	*T*_g_ (°C)	*T*_m_ (°C)	Δ*H*_m_ (J g^−1^)
PHB	100	—	—	1.84	2.2	3	175.9	91.5
PHBV	80.9	19.1 (HV)	—	1.53	2.1	−0.4	160.5	78.4
PHBHHx	81.2	18.8 (HHx)	—	5.68	1.7	−0.6	103.1	75.4
PHBA	84.8	9.4 (HO)	5.8	1.15	3.4	−5.9	133.4	67.1

The biodegradability of these polyesters was evaluated by weight loss (mg cm^−2^), considering typical surface erosion resulting from enzymatic hydrolysis. It was found that the order of biodegradability (poly(3-hydroxybutyrate – 19 mol% 3-hydroxyvalerate) (PHBV) > poly-3-hydroxybutyrate (PHB) > poly(3-hydroxybutyrate – 19 mol% 3-hydroxyhexanoate) (PHBHHx) > poly(3-hydroxybutyrate – 15 mol% 3-hydroxyalkanoates) (PHBA)) did not correlate with the *T*_m_, *T*_g_, crystallinity and molecular weight but with the side-chain length of comonomers (PHB > PHBV > PHBHHx > PHBA). In general, the biodegradation rate increased with decreasing side-chain length (Fig. 18), except for PHB (the homopolymer), which was an outlier from this trend. Therefore, the authors proposed that the structural effect, *i.e.* length of side-chain in this case, was the predominant factor determining enzymatic hydrolysis of PHA with intermediate chain length. The side-chain length was considered to affect the hydrolysis in two ways: the depolymerase used in this study only show affinity to PHA with short side-chain, and the higher hydrophobicity of the medium side-chain prevents the access of the enzyme to the crystalline region of the PHA with short side-chains.

## 5. Concluding remarks and outlook

Considering the predicted growth in plastic demand and the global targets to reduce CO_2_ emission, it is essential that we initiate a transition to substitute fossil-based plastic feedstock for renewable alternatives. Polyesters allow for a broad selection of feedstock and building blocks (monomers) from biobased and other renewable resources. Furthermore, polyesters can be tuned to provide excellent physico-mechanical, thermal, barrier and biodegradation properties for a wide range of applications.

Currently, many plastics utilised on an industrial level accumulate in the environment, and some of these plastics proceed to release microplastic particles; an improvement in waste management is necessary. The three R's (reduce, reuse and recycle) are a well-known framework to deal with these issues. However, (single-use) plastics are important for sanitation and food safety. Recycling heavily relies on collection and recycling infrastructures, which are lacking in many countries, especially in the most populated parts of the world. Therefore, biodegradability should be combined with the three R's.

As a result, next-generation plastics should be either closed-loop recyclable when the infrastructure is available, or designed to degrade completely (*e.g.* mineralised) over time when ending up in the environment. This necessitates the incorporation of biodegradability as a design feature for applications with an expected short lifetime or collection challenges, such as (food) packaging, mulch films, fishery material and disposable medical items. Furthermore, we should ensure that biodegradable plastics are not advertised in a way that could encourage littering.

Polyesters have the potential to be recycled by both mechanical and chemical processes (Table S1). Therefore, using biodegradable polyesters could help to smooth the transition from the current scenario, where plastics are leaked to the environment, to a future circular economy, where plastics are largely recycled.

The hydrolysable ester bonds offer potential for biodegradation in the environment, especially for polyesters that are susceptible to non-enzymatic hydrolysis at a moderate rate at ambient temperature. Their hydrolysis does not rely on enzymes, so degradation is likely to still take place under unfavourable conditions for biodegradation, such as in the deep sea. Therefore, non-enzymatically hydrolysable polyesters contribute to reducing plastic accumulation in the environment, especially for marine environments. Commercialisation of such polyesters may also reduce the effects of MNPs.

Large quantities of biodegradable polyesters entering the environment can be considered as an input of carbon, which might affect the carbon and nitrogen dynamics.^101–103^ Some microbial species could be enriched by biodegradation and result in the change of microbial communities, which might decrease the biodiversity.^102^ For instance, lactic acid could facilitate the growth of lactic acid bacteria, which will generate toxic substance for other microbes and fungi.^104^ Therefore, qualitative and quantitative investigation into the biodegradation products and their interactions with (micro)organisms and the environment is necessary.

Although the sensitivity to non-enzymatic hydrolysis will reduce the service life of the polymers/plastics (“plastics with an expiry date”), the benefits of environmental biodegradation not requiring specific hydrolase enzymes should outweigh the disadvantages. For many applications, we must consider the trade-off between convenience and reduction in environmental risk. Similar to the variety of thermal and mechanical properties, polyesters also show great potential for designing their biodegradability by selection of building blocks, even though this does require the development of novel synthetic approaches and copolymerisation. Generally, incorporation of cyclic monomers as rigid building blocks, including aromatic and aliphatic compounds, improves certain thermomechanical properties of polyesters. For hydrocarbon-based monomers, this typically leads to a regression in the biodegradation and/or non-enzymatic hydrolysis rate, which becomes worse with higher molar ratio of these cyclic monomers. This could be the result of (1) the decrease in the flexibility of the polymer chain, which decreases the probability of hydrolysis *via* enzymes with deep active-sites; and/or (2) the steric hindrance/hydrophobicity of the cyclic structure. Biobased isosorbide could be an alternative where the cyclic structure can provide good thermal (*i.e. T*_g_) and mechanical properties to polymers, while also leading to a high level of (bio)degradability.^75,87,89^

On the other hand, increasing the chain length, *i.e.* amount of methylene units within aliphatic building blocks for polyesters, could tune the polyester towards being more flexible (and elastomeric), while long chain (>C11) polyesters show significantly less biodegradability than polyesters with medium chain lengths. This may be attributed to the decrease in hydrophilicity. There is no clear trend in biodegradability for short- and middle-length chains. The shortest diacid, oxalic acid, shows sensitivity to hydrolysis. Additionally, (longer) side-chains can result in a raised hinderance to the hydrolysis of polyesters.

The development of novel polyesters from sustainable sources leads to a wide variety of potential compositions. The specific balance between thermal properties, mechanical properties, and biodegradability requires comprehensive investigation on the structure–biodegradability relationship.

Biodegradation is a complex process that may occur *via* multiple mechanisms at varying rates under different conditions. Given the influence of multiple factors on biodegradation, the use of machine learning could be beneficial for predictive modelling to offer a partially-quantitative approach. These models must consider both internal factors (such as polymer composition) and external factors (*e.g.* marine environment). This approach can also help identify the significance of factors affecting the biodegradability of a specific type of polyester under defined conditions.^105^ Consequently, it allows for preliminary screening to select promising polymer compositions suitable for specific applications (computational simulations) and provides control over these internal factors during the polymer design phase.

Moreover, ideally, with the advancement of artificial intelligence (AI) and the development of predictive models, users are allowed to set the requirements for biodegradability and conditions for usage and/or end-of-life, considering specific applications. AI can then assist in the design of (co-)polyester compositions.

However, it's important to emphasis that introducing a product to market entails many factors, and chemical design represents only one aspect within a multidisciplinary process. Biodegradability is just one factor in overall the design.^106^

To achieve this, we need a comprehensive database of well-characterized polyester biodegradation results under a specified environment. In addition to systematically recoding data, AI could be trained to extract information from published research articles. However, physically conducting experiments will always be required to validate the AI model's output, and to expand the database, which will therefore improve the model.

As biodegradation tests are time-consuming, it is important to employ high-throughput platforms for efficient research into the biodegradability of a polymer.^60^ This allows for study of the biodegradability of novel polymers with various compositions in a time-efficient way. Therefore, expanding the scope of research into novel materials already in the early stage at a limited cost.

## Abbreviations

CCU: Carbon capture and utilisation
FDCA: 2,5-Furandicarboxylic acid
BD: 1,4-Butanediol
BOD: Biochemical oxygen demand
DCA: Dicarboxylic acid
DSC: Differential scanning calorimetry
GA: Glycolate/glycolic acid
GA: Glycolic acid
HB: 3-Hydroxybutyrate
HDO: 1,6-Hexanediol
IS: Isosorbide
IUPAC: International Union of Pure and Applied Chemistry
LA: Lactic acid
*M*_n_
: Number average molecular weights
MNPs: Micro- and nano plastics
PBADs: Poly(butylene *n*-alkylene dicarboxylate)
PBAT: Poly(butylene adipate-*co*-terephthalate)
PBDd: Poly(butylene dodecanedioate)
PBF: Poly(butylene furandicarboxylate)
PBFGA: Poly(butylene furandicarboxylate-*co*-glycolate)
PBIS: Poly(butylene-*co*-isosorbide) succinate
PBS/PBS(u): Polybutylene succinate
PBSA: Poly(butylene succinate-*co*-butylene adipate)
PBTF: Poly(1,4-butylene 2,5-thiophenedicarboxylate)
PBUd: Poly(butylene undecanedioate)
PC: Polycarbonate
PE: Polyethylene
PEF: Poly(ethylene 2,5-furandicarboxylate)
PET: Poly(ethylene terephthalate)
PHA: Polyhydroxyalkanoates
PHB: Polyhydroxybutyrate
PHBA: Poly(3-hydroxybutyrate – 15 mol% 3-hydroxyalkanoates)
PHBHHx: Poly(3-hydroxybutyrate – 19 mol% 3-hydroxyhexanoate)
PHBV: Polyhydroxybutyrate-*co*-hydroxyvalerate
PIS: Poly(isosorbide succinate)
PISOX/PISOX100: Poly(isosorbide oxalate)
PLA: Poly(lactic acid)
PLGA: Poly(lactic-*co*-glycolic acid)
POBC: Polyoxabicyclates
POP: Persistent organic pollutant
PP: Polypropylene
PS: Polystyrene
*T*_g_
: Glass transition temperature
Three R's: Reduce, reuse and recycle
*T*_m_
: Melting temperature
TPA/T: Terephthalic acid

## Conflicts of interest

There are no conflicts of interest to declare.

## Supplementary Material

GC-026-D3GC04489K-s001
