# Supplementary material for: Polyester biodegradability: importance and potential for optimisation
Source: Green Chem. 2024 Mar 5;26(7):3698–716. doi: 10.1039/d3gc04489k (PMC10986773; doi:10.1039/d3gc04489k)
Supplement: GC-026-D3GC04489K-s001 [file GC-026-D3GC04489K-s001.pdf]

Electronic Supplementary Material (ESI) for Green Chemistry.
This journal is © The Royal Society of Chemistry 2024

Supporting Information

Polyester biodegradability: importance and potential for optimization

Yue Wang,[a, b] Robert-Jan van Putten,[c] Albert Tietema,[b] John R. Parsons [b] and Gert-Jan M. Gruter*[a,c]

[a] van 't Hoff Institute for Molecular Sciences (HIMS), University of Amsterdam, Science Park 904, 1098 XH Amsterdam, the Netherlands.

[b] Institute for Biodiversity and Ecosystem Dynamics (IBED), University of Amsterdam, Science Park 904, 1098 XH Amsterdam, the Netherlands.

[c] Avantium Support BV, Zekeringstraat 29, 1014 BV Amsterdam, the Netherlands; robert-jan.vanputten@avantium.com

* Correspondence: g.j.m.gruter@uva.nl.

Table S 1 Overview of polyesters discussed and their potential recyclability. n.a. = not applicable (not biodegradable); n.i.a. = no information available.

Section	Polyester	Molecular structures	Biodegradation methods – Parameters	Biodegraded products	Reference	Potential recyclability[a]
Aromatic diacid	PET	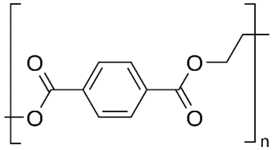	Composting (58 °C, 270 d) – CO_2	n.a.	Fig.8[85]	- Mechanical (most commonly), chemical, biological[24,77,78,107]
	PEF	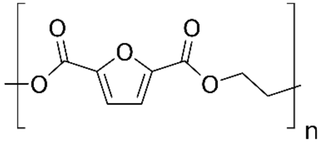	Composting (58 °C, 450 d) – CO_2	CO_2,	Fig.8[85]	- Mechanical (compatible with current PET recycling systems), chemical, biological[78,85,107,108]
	PBAT	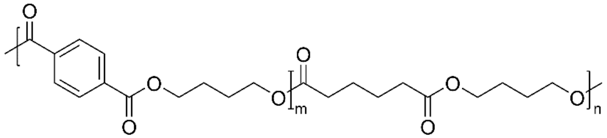	Composting (50 °C) – Mass loss; Enzymatic hydrolysis (37-55 °C, 1 – 18 d) – Titration	n.i.a.	Fig.5 & Fig.6[62]	- Mechanical (for PLA/PBAT[76]), chemical[109], biological[79]
			Enzymatic hydrolysis (30 °C, 20 h) – Mass loss	n.i.a.	Fig.7[65]	
	PBFGA	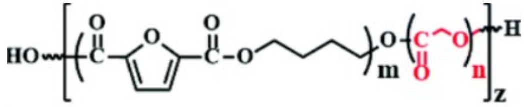	Non-enzymatic (35 d) & enzymatic hydrolysis (70 d) (37 °C) – Mass loss	n.i.a.	Fig.9[86]	- n.i.a.<br>- *PBF: chemical[110]

	PBIS<br>(PIS/PBS/<br>PBSA)	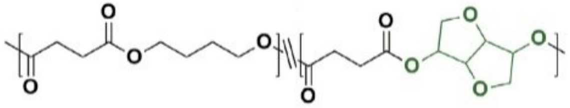<br>PBIS copolyester	Non-enzymatic & enzymatic hydrolysis (37 °C, 16 w) - Mass loss	n.i.a.	Fig.10[87]	- n.i.a.<br>- *PBS[76]: biological[111,112]<br>- *PLA/PBS: mechanical, chemical and biological[76,113]
Cyclic aliphatic diol<br>/<br>linear aliphatic monomer	PISOX	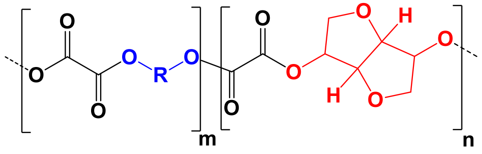<br>R listed below<br>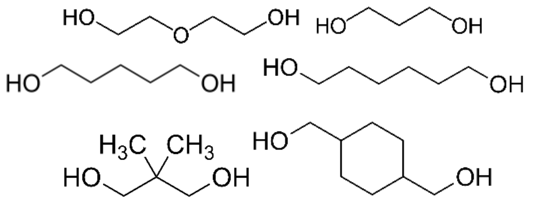<br>OX was replaced by TPA in one of the composition<br>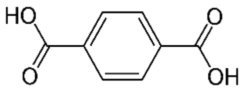	Biodegradation in soil (25 °C, 270 d), seawater and sediment (25 °C, 53 d) – CO_2;<br><br>Non-enzymatic hydrolysis (25 °C, 185 d) – Soluble monomers	CO_2;<br><br>monomers including oxalic acid, isosorbide and other co-diols	Fig.11[89,90]	- n.i.a.

Linear aliphatic monomer	PBAD	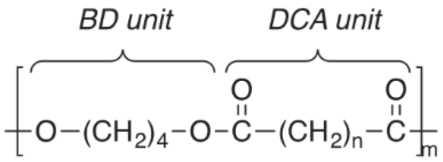<br>n = 2-10; n = 10-14	Biodegradation in an aqueous medium (supernatant of soil suspension as inoculum, 25 °C, 30 d) – O_2	CO_2	Fig.12[90]	- n.i.a
			Biodegradation in an aqueous medium (supernatant of soil suspension and PBAD degrading isolates as inoculum, 25 °C, 30 d) – O_2	n.a.	Fig.13[91,92]	
	POBC	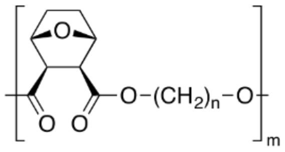<br>n = 2,3,4,6,8,10	Biodegradation in an aqueous medium (supernatant of soil suspension and PBAD degrading isolates as inoculum, 25 °C, 90 d) – O_2	CO_2	Fig.15[93]	- n.i.a
Branched monomer	PLGA	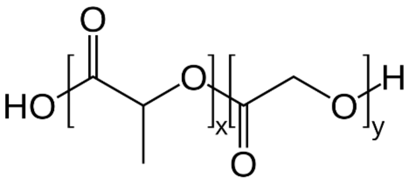<br>x= LA, y = GA	Non-enzymatic hydrolysis (37 °C, 7 w) – Mass loss, intrinsic viscosity	n.i.a.	Fig.16[98]	- *PLA: mechanical, chemical, biological[76,77,107,114]
			Biodegradation in soil (25 °C, 53 d) – CO_2;<br>Non-enzymatic hydrolysis (25 °C, 116 w) – Soluble monomers	CO_2;<br>GA、 LA	Fig.17[60]	

PHAs	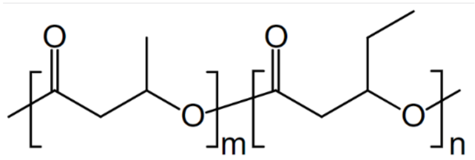<br>m =HB; n =HV; n is replaced by HHx and HO for other copolyesters<br>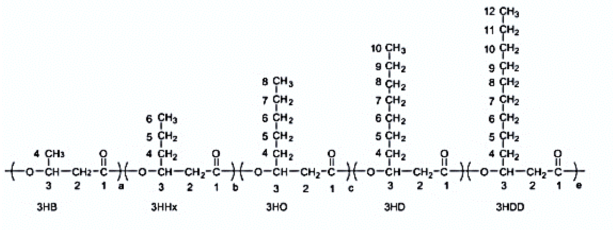 	Enzymatic hydrolysis (37 °C, 25 h) – <u>Mass loss</u>	HB monomer, dimers HB–HB, HV–HB, HV–HV, HB–HHx and HB–HB–HHx trimers	Fig.18[100]	- Biological[76,77,79,115]<br>- * PLA/PHAs: mechanical[76,116]

a. Chemical recycling specifically refers to depolymerization into monomers and or low molecular weight polymers, enabling subsequent polymerization; pyrolysis is not covered in this context.

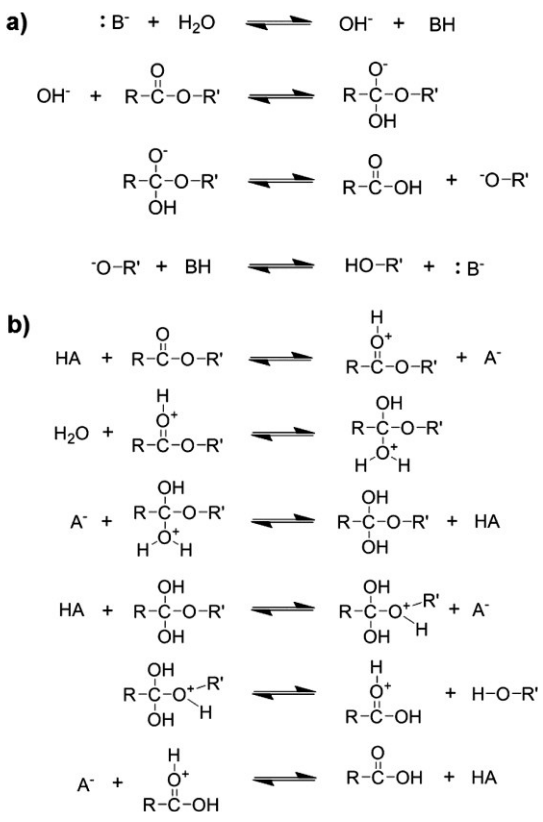

Figure S 1 Mechanism for (a) base-catalysed and (b) acid-catalysed hydrolysis of polyesters. Reprinted with permission from[64]. Copyright © 2018 American Chemical Society.
